# Assessment of the mind-body connection: preliminary psychometric evidence for a new self-report questionnaire

**DOI:** 10.1186/s40359-023-01302-3

**Published:** 2023-10-06

**Authors:** Kristen Van Bael, Michelle Ball, Jessica Scarfo, Emra Suleyman

**Affiliations:** Institute for Health and Sport, Victoria University, Melbourne, Victoria Australia

**Keywords:** Interoception, Emotion, Mind-body connection, Scale development, Reliability, Validity

## Abstract

**Objectives:**

While interoceptive self-report scales provide a foundation for measuring the mind-body connection, they variably consider other important factors that could influence interpretations of internal bodily sensations and perceptions related to mind-body integration. The proposed Body-Mind Connection Questionnaire (BMCQ) aimed to operationalise the notion that this construct involves three major components: (a) Interoceptive Attention, (b) Sensation-Emotion Articulation, and (c) Body-Mind Values.

**Methods:**

Following panel review and piloting with the target population, the developed BMCQ was evaluated in 316 participants (189 identifying as female) aged 18-50 (*M*_*Age*_=30.78), alongside established self-report measures of interoceptive sensibility, body awareness, sensory processing sensitivity, and alexithymia. We examined the BMCQ factor structure through exploratory factor analysis and analysed convergent and discriminant validity.

**Results:**

Exploratory factor analysis supported three scales of the BMCQ, which explained 54.03% of variance. Factor loadings (>0.44) and reliability indices (0.74 to 0.85) were acceptable. Inter-scale correlations suggested that the scales are distinct but related (*r*s=0.38 to 0.59). BMCQ scales were supported by convergent (*r*=0.33 to 0.67) and discriminant evidence (*r*s=0.01 to 0.39, *p* range n.s. to <.05).

**Conclusions:**

Preliminary psychometric properties indicate that the BMCQ is multidimensional and consists of three constructs that differentially relate to theoretically associated measures. Interoceptive Attention, Sensation-Emotion Articulation, and Body-Mind Values may serve as a basis for efficiently assessing the mind-body connection more holistically, which could be useful for developing interventions aimed at enhancing mind-body integration.

**Supplementary Information:**

The online version contains supplementary material available at 10.1186/s40359-023-01302-3.

## Introduction

A popular view of the mind and body is that they exist as distinct and separable entities. While some may experience their mind as qualitatively different to their body, mind-body dualism is not biologically plausible. Major advancements in neuroscience indicate that cognition is embodied [45], where continual interactions between the environment and the individual’s body and brain influence thoughts and feelings to facilitate situationally appropriate behaviour [6]. This recognition that the body and mind operate as a connected and integrated force has immense implications for physical and psychological health (e.g., [38, 43]).

Several processes emerge as exemplifying the mind-body connection, namely interoception and emotion. Interoception refers to the processes by which the nervous system anticipates, senses, interprets, integrates, and regulates signals originating from the body across unconscious and conscious levels [55, 77]. By extension, emotions represent psychological states that involve subjective experiences, physiological responses, cognitions, and expressive behaviours [48] that are associated with motivational reorientation [30]. Influential theories posit that emotions arise from the capacity to sense changes from within the body (e.g., [28]). As such, interoception forms a core component of emotion [5] and facilitates adaptive behaviour driven by emotional experience [27, 30].

Despite a growing appreciation of these functions underlying the mind-body connection, existing self-report measures in this area vary in the degree to which emotional processes are captured. What appears omitted amongst them is the consideration of broader values regarding the mind-body connection, which may provide additional insights into the role of holistic health and wellbeing perspectives in physical and psychological outcomes. Considering these factors, a new self-report measure of the mind-body connection incorporating these notions was developed.

### Interoception: a foundation of emotion and the mind-body connection

Maintaining desirable physiological states is critical for assuring survival and serves as the foundation of physical wellbeing [23, 30], which is enabled through interoception. Interoception is an iterative function, requiring the intricate interplay between the perception of bodily states (e.g., fatigue, hunger, thirst) and cognitive interpretation of such states to inform appropriate action [24]. Interoception is intimately tied to physiological regulation via homeostasis—the dynamic processes which maintain physiological integrity of the body at setpoints or within narrow ranges conducive to survival and optimum function [18, 24, 71]. Physiological signals arising from the body are projected directly to autonomic and homeostatic regions located in the spinal cord, brainstem, and thalamus for processing [9, 23]. Signals are then projected to a multitude of cortical and subcortical regions that regulate internal physiological systems (e.g., autonomic, immune, neuroendocrine), and represent sensations originating from the body [57].

Interoceptive signals continuously interact with cognitive models comprising past experiences and internal and external environmental regularities (e.g., [9, 83]), which informs experience and interpretation, and motivates regulatory behaviour [24, 25]. This whole-brain network coordination enables the redistribution of physiological and psychological resources to meet individual needs, particularly in the face of environmental challenges [27], and accordingly exemplifies a mind-body connection function.

A longstanding theoretical tradition describes emotions as inexorably linked to changes arising from within the body through interoception (e.g., [5, 29, 53, 80]), where both functions are tied to the cognitive interpretation of changes occurring from within the body [27]. A burgeoning body of neuroanatomical evidence substantiates such views, indicating that neural regions activated during interoceptive processing overlap with those implicated in motor coordination (e.g., [6]), emotional experiences [5, 24, 26, 61, 82, 101] and emotion regulation [97, 104]. In line with such evidence, it is tenable that interoception plays a vital role in motivating behaviours that seek to restore or maintain bodily balance and wellbeing, based upon how—or whether—one affectively interprets the sensation in context [24, 26, 89]. Despite these links, the degree to which emotional functions are captured in existing assessments of the mind-body connection is insufficient.

### Interoceptive dimensions and assessment methods

It is now accepted that conscious interoception, which forms the foundation of emotional processes, is multidimensional [55]. Various frameworks exist (e.g.,  [55, 67, 72, 90]), although the three-dimensional model proposed by Garfinkel and colleagues [47] remains widely endorsed [35]. These domains include interoceptive accuracy (IAcc), interoceptive awareness, (IAw), and interoceptive sensibility (IS). IAcc pertains to the capacity to accurately detect internal bodily sensations, which is typically assessed through objective performance. IAw refers to the metacognitive awareness of accuracy—conceptualised as confidence in the accurate monitoring of internal bodily sensations. Quantification involves the combination of objective interoceptive performance and a subjective appraisal of this. Importantly, tasks proposed to capture accurate monitoring and appraisals implicitly involve bias, due to the role of purposeful top-down directed attention toward an interoceptive channel [2].

On the other hand, IS is a purely subjective dimension, encompassed by the dispositional inclination to possess an internal focus and be interoceptively cognisant [47]. Typically, IS is operationalised through self-report scales that capture trait-based functions [35]. The IS domain has notably been conceptualised as an expression of a high-level model or overarching ‘belief’ which influences how one tends to interpret interoceptive signals and infers the causes of them [27].

While acuity in accuracy and awareness is crucial for maintaining optimal functioning and facilitates adaptive behaviour [94], the subjective component of interoception is complex and nuanced [55]. Although any subjective assessment inherently involves bias, strongly held beliefs regarding interoceptive abilities may influence overall interpretation of bodily sensations [27, 90] and subsequently guide the enactment of behaviours aimed at addressing equilibrium and wellbeing.

In interoceptive research, the dimensions of interest are primarily those utilising behavioural performance measures (i.e., IAcc and IAw; [55]). Assessments for these dimensions chiefly involve heartbeat tracking (heartbeat discrimination task; [98]) or counting [81], although other physiological tests are utilised (e.g., water load task for gastrointestinal sensitivity, respiratory resistance load detection, [27]). Whilst these are traditionally measures of IAcc and IAw, they assess a singular bodily channel and are not generalisable [39]. Furthermore, the psychometric properties of predominant cardiac measures have been subject to much scrutiny. A major criticism pertains to the influence of subjective prior beliefs on performance (e.g., [34, 79, 102]), which limits reliability, validity, and interpretation. Furthermore, when data have been aggregated for meta-analysis, performance on the heartbeat counting task does not appear to be significantly associated with major indicators of wellbeing, including trait anxiety, depression, or emotion deficits [36].

By contrast, subjective beliefs regarding the degree to which individuals are interoceptively cognisant appears to influence clinical indicators, including depression severity [37] suicidal ideation amongst persons diagnosed with eating disorders [70], and contributes to distorted bodily beliefs amongst high and low physical symptom reporters and persons with somatoform disorders [42]. As such, self-report measures pertaining to beliefs and interpretations of bodily sensations may be more suitable indicators of clinical status than behavioural measures [90]. Psychological interventions are being developed to target facets of subjective interoception to improve global bodily awareness and enhance utilisation of internal cues for self-regulation in disorders characterised by interoceptive and emotional dysfunction [14]. However, self-report measures and conceptualisations of this construct vary in the degree to which emotion is considered.

### Limitations of interoceptive self-report scales

Relative to IAcc and IAw, interoceptive self-report scales capture appraisals across multiple channels by nature of definition and design. There are many existing tools, which assess a range of beliefs available to conscious access. Trevisan and colleagues [94] (see also [67]) surmised that scales generally assess: i) self-reported tendency of attention toward bodily signals relevant to homeostatic needs (e.g., thirst, hunger) and to some degree, emotional arousal, and ii) self-perceptions of accuracy in the discrimination and interpretation of such signals.

Through systematic review, Desmedt et al. [35] identified that the Body Awareness scale of the Body Perception Questionnaire (BPQ, [73]), the Body Awareness Questionnaire (BAQ, [85]), and the Multidimensional Assessment of Interoceptive Awareness (MAIA, [65, 66]) are most frequently used in research. The Body Awareness scale of the BPQ is a unifactorial measure of hypersensitivity and maladaptive attention toward autonomic nervous system structures and accompanying sensations [58, 63], but does not explicitly capture elements of emotional arousal nor appraisal. Conversely, the BAQ is a unifactorial measure of self-reported attentiveness to normal non-emotive body processes, including adaptive sensitivity to body cycles and rhythms, and does not empirically relate to affective experience [103]. By contrast, the MAIA comprises eight scales, assessing subjective interoceptive awareness in various domains. Table 1 presents an overview of these.

**Table 1:** MAIA-2 Scales, abbreviations and descriptions

Scale	Abbreviation	Description
Noticing	Not.	Awareness of uncomfortable, comfortable, and neutral body sensations
Not-Distracting	ND	Tendency not to ignore or distract oneself from sensations of pain or discomfort
Not-Worrying	NW	Tendency not to worry or experience emotional distress with sensations of pain or discomfort
Attention Regulation	AR	Ability to sustain and control attention to body sensations
Emotional Awareness	EA	Awareness of the connection between body sensations and emotional states
Self-Regulation	SR	Ability to regulate distress by attention to body sensations
Body Listening	BL	Active listening to the body for insight
Trusting	Trust.	Experience of one’s body as safe and trustworthy

Amongst MAIA scales and within the overarching framework, emotion is set out to be explicitly captured [65, 66]. For instance, the EA scale measures the awareness of the connection between body sensations and emotional states. Further, regulatory behaviours driven by bodily interpretation are measured, per AR, SR, and BL items [14]. Together, these scales are proposed to facilitate measurement of adaptive attentional styles and regulatory functions underpinning IS than existing measures, such as the BPQ [63].

Prima facie, the MAIA is a comprehensive, multidimensional measure of self-reported interoception. Emerging evidence suggests that the MAIA might instead measure three constructs—not eight. Ferentzi et al. [39] identified that the MAIA is comprised of a general interoception factor, consisting of Not., AR, EA, SR, BL, and Trust. subscales, with ND and NW subscales emerging as distinct and unrelated factors. These findings have been corroborated by Desmedt et al. [35] and extended by Todd et al. [92], suggesting that a summary score comprising these six MAIA scales is a pragmatic measure of IS. Whilst interoceptive self-report scales such as the MAIA conceptually consider mind-body integration, these aspects could be enhanced through further scrutiny of related constructs that may improve how the mind-body connection is currently assessed via self-report—primarily through interoceptive self-report scales.

Interoceptive attention serves as a crucial function underpinning the interpretation of interoceptive signals [90]. Indeed, existing self-reports emphasise this process; elements of interoceptive attention toward bodily signals relevant to homeostatic needs are certainly measured within the BPQ, BAQ, and MAIA. The BPQ appears to assess hypersensitivity, whereas the MAIA and BAQ capture adaptive attentional functions related to interoceptive processing. However, interoceptive attention has been described as involving the capacity to direct attentional resources toward internal bodily sensations that can be captured in bottom-up, stimulus-dependent or top-down, purposeful manners [56]. If attention is understood to be stimulus-driven or goal-directed [21], then measurement of subjective interoceptive attention could alternatively entail the habitual allocation of resources to a sensation if suddenly experienced due to homeostatic perturbation or purposefully contemplated. In line with such views, items amongst these interoceptive self-report scales seem to lack the explicit measurement of such notions pertaining the differing ways that attentional resources can be directed or allocated toward internal sensations.

Furthermore, alexithymia is of worthy consideration in reviewing how emotion could be captured in a mind-body connection self-report, given its characterisation as “the quintessence of impairment of mind-body connection” [31], p. 2). Alexithymia is underpinned by deficits in identifying feelings and differentiating between feelings and bodily sensations associated with emotional arousal, describing feelings, and an externally oriented thinking style, by which there is a preoccupation with details and features of the external environment [86]. Alexithymia is of clinical significance and is associated with subjective health and wellbeing perceptions, including somatisation (i.e., the tendency to experience and report physical symptoms due to emotional distress, [60]), physical symptom severity [78], and health-related quality of life [54, 62]. Various studies report a link between interoception and alexithymia (e.g., [15, 46, 93]), with findings suggesting that alexithymia is the culmination of poor interoceptive perception across multiple channels [67] and even convergence [44, 96]. Both hypersensitivity and hyposensitivity toward bodily sensations may exacerbate poorer emotional articulation, thus impinging on effective mind-body communication [1, 10, 68]. Whilst MAIA items seem to capture the ability to identify the connection between body sensations and emotional states, the additional ability to describe that link is not assessed. Furthermore, items are only suggestive of directing attention internally, rather than explicitly measuring preferences for an internally- or externally oriented focus. Greater incorporation of the capacity to articulate the emotional meaning of internal bodily sensations may enhance measurement of emotion, alongside explicit measurement of preferences for possessing an internal focus. Conceptualised together, these may enrich mind-body self-reports and enable the development of more targeted interventions aimed at improving adaptive cognitions, emotions, and behaviours in clinical populations.

Furthermore, an embodied sense of self reflects a high-order function that can encompass facets of regulatory behaviours guided by bodily cues and trust in body sensations [65], which may contribute to overall health and wellbeing [38]. Conversely, conceptualising the mind as distinct from the body may lead to poorer wellbeing, particularly in line with evidence suggesting that dualistic views are linked reduced health-related behaviours (e.g., [16, 43]). A recent hierarchical interoceptive framework specifies that an ‘attribution of sensations’ dimension represents the zenith of sensation interpretation [90]. Considering that sensations may be interpreted as benign, ambiguous, or threatening, whereby clinical groups may be negatively biased in their interpretations, such attributions may be a consequence of overarching mind-body beliefs. As such, whether one values physical and mental wellbeing may yield additional insights into the sense of connectedness with both mind and body. Many existing interoceptive self-reports capture attitudinal tendencies pertaining to bodily cues, such as BAQ and the MAIA. However, broader attitudes pertaining to whether physical and mental wellbeing are important and prioritised are not measured.

In sum, prevalent interoceptive self-report scales vary in the concurrent measurement of emotional abilities that could relate to how individuals believe they detect and attend to specific sensations. In addition, there appears variable consideration of broader values pertaining to the mind-body connection and the importance of wellbeing. It is proposed that omission of these factors impinge upon the measurement of an individual’s perceived connection to their body and mind. For these reasons, the Body-Mind Connection Questionnaire (BMCQ) was developed to attempt to address limitations identified with existing self-report measures so as to enable clearer identification of whether individuals exhibit a connection with mind and body.

### Scale construction

In accordance with suggested guidelines for scale development (e.g., [11]), key domains involved in the mind-body connection were conceptualised by the research team. This guided item generation for the BMCQ, and involved: (a) review of theory, literature and pre-existing scales related to interoception and emotion to ascertain key constructs for mind-body connection measurement,(b) item generation of positively- and negatively-worded items based on reviews; (c) screening for item redundancy relative to existing scales; (d) determining measure structure (e.g., item complexity, response format), (e) panel and target population review of drafted items; and (f) synthesis and integration of feedback and assembly of measure for field test.

Following review, three key domains were identified, and proposed to be qualities informing one’s holistic connection to their body and mind. These were conceptualised as ‘Interoceptive Attention’, ‘Sensation-Emotion Articulation’, and ‘Body-Mind Values’. A total of 59 items were generated for these domains. Specifically, Interoceptive Attention items followed a theoretical grounding in foundational capacities, whereby these reflected non-biased, selective attentional capacities, as other functions (e.g., sustained, divided) are captured in the MAIA. Eighteen items were generated for this domain. While ‘Emotional Awareness’ is captured in the MAIA, BMCQ items generated for the Sensation-Emotion Articulation domain drew upon alexithymic characteristics and constituents, following recent evidence showing the presence of a latent interoceptive factor in alexithymic assessment tools [44] coupled with indication that MAIA items only seem to measure identification of the link between sensation and emotion. Items were formulated to represent the inverse of alexithymic characteristics. Twenty-three items were generated for this domain. Lastly, Body-Mind Values item generation was guided by identification that existing assessment tools capturing attitudinal facets of mind-body integration but primarily pertain to bodily cues (e.g., MAIA, [65]), with psychological cues not explicitly captured amongst items. Furthermore, that broader endorsement of mind and body are connected entities is not captured by existing tools. For this domain, 19 items were generated.

All items were screened for redundancy. If an item replicated items from existing questionnaires, it was removed. Next, remaining items were assessed for several factors, including grammatical complexity and technical jargon, and were rephrased to increase understanding or readability where necessary. Following these processes, the BMCQ was reduced to a 22-item pool. With respect to response scale ratings, it was agreed that clearly defined 7-point scales would enable respondents a greater degree of distinction in endorsement of BMCQ items. This was determined, as reliability and validity are better retained, and biases influencing responses (e.g., extreme response, acquiescence) are mitigated [59].

Four researchers with expertise in biological psychology and familiarity with interoception independently provided feedback on face validity, theoretical coherence, wording, and item clarity. Where there were differences in opinion, open discussions were held to explain perspectives which facilitated consensus being reached. Following discussions, several items were refined for clarity; the item pool was collectively deemed to be relevant to the hypothesised constructs. This process resulted in a retained 22 item pool to assess three constructs: Interoceptive Attention (five items regarding the ability to direct attentional resources toward interoceptive stimuli in purposeful and spontaneous manners), Sensation-Emotion Articulation (seven items related to the capacity to identify and describe internal bodily changes in emotional contexts and a preference for the internal environment), and Body-Mind Values (10 items reflecting beliefs in mind-body integration and perceived importance of wellbeing).

The BMCQ was then administered to 25 individuals known to the researchers to assess face validity from the perspective of respondents. This is recommended to determine whether target respondents interpret the items as intended [11]. Due to implemented COVID-19 restrictions, participants’ mental processes and experiences responding to the BMCQ were provided in written form. This was hosted on Qualtrics (Qualtrics, Provo, UT [76]). The study link was circulated through social media platforms (e.g., Facebook) and amongst members of the research team. Target respondents provided their insights as they completed the questionnaire, inclusive of clarity of items or aspects of experience not considered. Throughout the online study, textboxes were provided after each section for participants to record their experiences and observations.

**Fig. 1:**
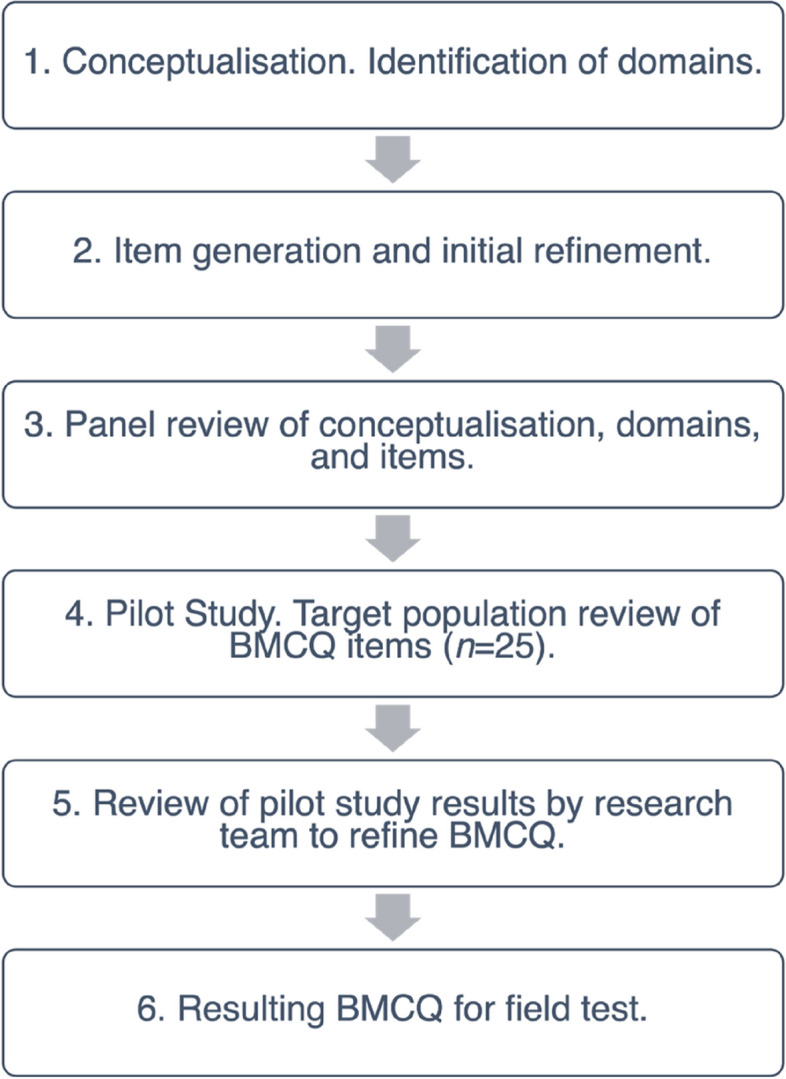
Iterative Sequence of Development of the BMCQ

The research team synthesised and reviewed these results. This included assessment of feedback for each item, and whether they yield skewed responses through review of means and standard deviations. No problematic items were identified. The 22-item BMCQ was retained and administered for field testing. Figure 1 contains an overview of stages involved in scale development.

## Method

### Participants

An *a priori* sample size of 220 participants for the field test was determined to be the absolute minimum for this study, as this would provide a ratio of 10:1 respondents per item [11]. Scale development and item reduction are contingent upon larger samples in order to produce more stable factor solutions [91]. Accordingly, a total of 417 participants aged 18 to 50 were recruited using the Prolific recruitment service. To be included in the study, participants were required to be:

1. Aged 18-50, to limit the effects of aging on physical health, including beliefs and practices [32]
2. Fluent in the English language
3. Reside in Australia, Canada, New Zealand, the United Kingdom, or the United States
4. Free of a current chronic pain condition (e.g., fibromyalgia, severe arthritis), to limit the effects of chronic pain on subjective processing of homeostatic factors [88].

Except for a current diagnosis of a chronic pain condition, there were no specific criteria to exclude individuals with a current diagnosis of other physical or mental health conditions. Participants were requested to disclose whether they were currently diagnosed with a physical and/or psychiatric condition, as previous literature indicates an association with altered interoceptive and emotional functioning (e.g., [55]). A total of 119 participants advised of current physical and/or mental diagnoses. Multiple conditions were self-reported. Eighteen participants reported physical conditions, whereas 101 participants disclosed of at least one current psychiatric diagnosis, including anxiety-related, depressive, feeding and eating, and neurodevelopmental disorders. Table S1 of the supplemental material provides an overview of all self-reported diagnoses. As psychiatric conditions can differentially influence interoceptive and emotional processing [56], these participants were excluded. Subsequently, 316 participants (*M*_*Age*_=30.78, *SD*_*Age*_=8.34) were included in the study. Table 2 contains additional demographic characteristics of the included sample.

**Table 2:** Demographic characteristics of the sample with no self-reported psychiatric diagnosis (*N=*316)

Characteristic	*N* (%)
Age	
18-19	28 (8.9%)
20-29	119 (37.7%)
30-39	117 (37.0%)
40-50	51 (16.1%)
Gender Identity	
Man or male	124 (39.2%)
Woman or female	189 (59.8%)
Another term (e.g., non-binary)	2 (0.6%)
Prefer not to answer	1 (0.3%)
Country of Residence	
Australia	9 (2.8%)
Canada	26 (8.2%)
New Zealand	8 (2.5%)
United Kingdom	225 (71.2%)
United States	48 (15.2%)
Level of Education	
Year 10 or lower	6 (1.9%)
Year 12	121 (38.3%)
Bachelor’s Degree	111 (35.1%)
Honours	15 (4.7%)
TAFE or vocational training	11 (3.5%)
Masters	41 (13.0%)
PhD or Doctorate	6 (1.6%)
Graduate Certificate	5 (1.6%)

Other indicators of health and wellbeing were also requested, including body mass index (BMI), smoking status, alcohol consumption, engagement in sport and exercise, yoga, and mindfulness and meditation practice. Table 3 contains this information.The sample were primarily non-smokers that infrequently consumed alcohol and regularly engaged in sport and/or exercise, and meditation and mindfulness practice. On average, the sample reported a BMI that would be classified as overweight (i.e., ≥25; [99]), although this was calculated based on self-reported height and weight (Table 3). Overall, the sample appeared to endorse relatively healthy characteristics and practices.

**Table 3:** Overview of health and wellbeing characteristics of the sample (*N=*316)

Characteristic	*N (%)*	*M (SD)*
Body Mass Index (BMI)^a^	296 (93.7%)	27.27 *(8.53)*
Underweight (<18.5)	10 (3.2%)	
Normal (18.5-24.9)	136 (43.0%)	
Overweight (25.29.9)	76 (24.1%)	
Obese (30+)	74 (23.4%)	
Smoking Status		
Smoker	33 (10.4%)	
Non-Smoker	283 (89.6%)	
Alcohol Consumption		
0-1 times per week	224 (70.9%)	
1-2 times per week	56 (17.7%)	
2-3 times per week	19 (6.0%)	
3-4 times per week	7 (2.2%)	
4 or more times per week	10 (3.2%)	
Sport or Exercise Engagement		
Yes (Hours per week)	208 (65.8%)	3.05 *(2.11)*
No	108 (34.2%)	
Yoga Practice		
Yes (Hours per week)	52 (16.5%)	0.97 *(1.64)*
No	264 (83.5%)	
Meditation and Mindfulness Practice		
Yes (Hours per week)	75 (23.7%)	1.23 *(1.30)*
No	241 (76.3%)	

^a^Calculated based on self-reported weight and height

## Materials

To test for convergent and discriminant validity, a questionnaire battery of several published measures sharing theoretical relatedness to the mind-body connection construct was assembled. This included the BPQ, BAQ, MAIA, Perth Alexithymia Questionnaire (PAQ), and the Highly Sensitive Person Scale (HSPS). Table 4 summarises these measures, score interpretation, ranges, means and standard deviations, and Cronbach’s alpha coefficients for the present sample.

**Table 4:** Score interpretation, possible and observed ranges of scores, means, standard deviations, and internal consistency reliability for validity measures (*N=*316)

Validity Measures	Interpretation	*N*	Possible Range	Observed Range	*M*	*SD*	Skewness	Kurtosis	Alpha
MAIA		313	0.00-5.00						
Not.	Higher scores reflect greater awareness of uncomfortable, comfortable, and neutral body sensations			0.00 - 5.00	3.28	0.93	-0.45	0.24	0.79
ND	Higher scores reflect a tendency to not ignore or distract oneself from sensations of discomfort and pain			0.00 - 5.00	1.97	0.96	0.42	0.38	0.88
NW	Higher scores reflect a tendency not to worry or experience emotional distress with sensations of pain or discomfort			0.00 - 4.80	2.56	0.89	-0.10	-0.06	0.78
AR	Higher scores reflect greater ability to sustain and control attention to body sensations			0.43 - 4.86	2.72	0.88	-0.05	-0.48	0.88
EA	Higher scores reflect greater awareness of the connection between body sensations and emotional states			0.20 - 5.00	3.33	0.97	-0.46	-0.16	0.85
SR	Higher scores reflect greater tendency to regulate distress by attention to body sensations			0.00 - 5.00	2.71	1.03	-0.31	-0.34	0.85
BL	Higher scores reflect actively listening to the body for insight.			0.00 - 5.00	2.45	1.15	-0.05	-0.56	0.85
Trust.	Higher scores reflect greater appraisals of the body being safe and trustworthy			0.00 - 5.00	3.12	1.15	-0.32	-0.49	0.89
BAQ	Higher scores reflect greater bodily awareness in non-emotive contexts	309	18-126	29 - 119	79.48	16.18	-0.23	0.02	0.87
BPQ	Higher scores reflect hypersensitivity toward sensations	313	26-130	26 - 130	71.69	21.6	0.41	-0.37	0.95
PAQ		313							
DIF	Higher values indicate greater difficulty identifying, understanding, and differentiating between one’s own positive and negative feelings.		8-56	8-56	24.83	10.03	0.23	-0.52	0.9
DDF	Higher values indicate greater difficulty describing and communicating one’s own positive and negative feelings.		8-56	8-56	29.06	10.24	-0.03	-0.32	0.9
EOT	Higher values indicate greater tendency to not focus attention on one’s own emotions (negative and positive).		8-56	8-50	26.45	9.91	0.12	-0.60	0.89
PAQ- Total	Higher values reflect greater levels of overall alexithymia; difficulty focusing attention on and appraising one’s own feelings (negative and positive).		24-168	26-162	80.35	26.9	0.04	-0.28	0.95
HSPS		313							
EOE	Higher values indicate greater ease of excitation		12-84	12-83	55.58	11.58	-0.18	0.05	0.84
AES	Higher values indicate greater sensitivity to aesthetic stimuli		7-49	7-49	31.1	6.57	-0.18	0.13	0.72
LST	Higher values indicate lower sensory threshold		6-42	6-41	20.81	7.26	0.30	-0.30	0.76
HSPS-Total	Higher scores indicate greater global sensory processing sensitivity		27-189	27-181	115.49	22.86	0.11	0.39	0.9

### Body-Mind Connection Questionnaire (BMCQ)

The BMCQ administered for field testing consisted of 22 items pertaining to Interoceptive Attention, Sensation-Emotion Articulation, and Body-Mind Values. Participants rated how applicable each statement is to them generally on a scale ranging from *not at all true of me* (1) to *very true of me* (7). Items were scored such that higher scores represented greater capacities in connecting mind with body. The 22-item version is provided in the Supplemental Material. Additional details regarding scale refinement, reliability, and construct validity are outlined in later sections.

### Multidimensional Assessment of Interoceptive Awareness, Version 2 (MAIA-2 [66])

The MAIA-2 is a 37-item self-report measure of interoceptive body awareness on 8 dimensions: 1) Noticing (MAIA-Not.); 2) Not-Distracting (MAIA-ND), 3) Not-Worrying (MAIA-NW), 4) Attention Regulation (MAIA-AR), 5) Emotional Awareness (MAIA-EA), 6) Self-Regulation (MAIA-SR), 7) Body Listening (MAIA-BL), 8) Trusting (MAIA-Trust). See Table 1 for MAIA-2 scale descriptions. Participants rate items on a 6-point scale ranging from *never* (0) to *always* (5), based on how often each statement applies to them generally in daily life. Scores are totalled and averaged for each subscale. Higher average scores reflect greater bodily awareness. MAIA scales demonstrate questionable to good internal consistency, where ɑ >0.65 for all subscales [66]. Incremental validity is shown through five subscales accounting for 41% of variance in trait anxiety [65].

### Body Awareness Questionnaire (BAQ [85])

The BAQ is an 18-item scale assessing self-reported attentiveness to normal non-emotive body processes, including sensitivity to body cycles and rhythms, changes in normal bodily functioning, and the ability to anticipate bodily reactions. Participants answer items on a seven-point Likert-scale, ranging from *not at all true about me* (1) to *very true about me* (7). Scores are summed, where higher values indicate higher body awareness. The BAQ shows good test-retest reliability (*r* = 0.72) and internal consistency reliability (ɑ = 0.79-0.83, [39, 40]). BAQ scores correlate with functional interoception, as measured by the MAIA (*r* =0.56, [35]).

### Body Perception Questionnaire (BPQ [15, 73])

The BPQ is a self-report measure of bodily awareness and autonomic reactivity. The BPQ consists of two subscales: Body Awareness and Autonomic Reactivity [17]. Studies examining IS utilise the Body Awareness subscale of the BPQ [36], which this study administered. This scale is unifactorial and consists of 26 items pertaining to subjective experiences of bodily sensations. Respondents rate how aware they are of these during most situations on a 5-point Likert-scale ranging from *Never* (1) to *Always* (5). A summary score is calculated, where higher values reflect hypersensitivity [58]. The *Body Awareness* subscale of the BPQ demonstrates excellent internal consistency (ω=0.92) and test-retest reliability (*r* =0.99*,* [17]). Convergent evidence indicates that Body Awareness relates to somatosensory amplification (*r*_*s*_=0.51) and stress reactivity (*r*_*s*_=0.57, [17]), but appears distinct from adaptive interoception, as measured by the MAIA (*r*=0.21) and attentiveness to bodily sensations, as measured by BAQ items (*r*=0.26, [35]).

### Perth Alexithymia Questionnaire (PAQ [74])

The PAQ is a 24-item measure assessing alexithymia. This includes difficulty identifying feelings (PAQ-DIF), difficulty describing feelings (PAQ-DDF), and externally oriented thinking (PAQ-EOT). Participants rate items according to how much they agree or disagree that the statement is true of them from *Strongly Disagree* (1) to *Strongly Agree* (7). The PAQ assesses both positive and negative emotion identification and description, as well as EOT. Separate subscales exist for positive and negative DIF and DDF. A total score is also calculated to indicate global alexithymia. As BMCQ Emotion-Sensation Articulation items reflect identification and description of positive and negative emotions, composite scores were computed to provide general DIF and DDF [74]. Higher PAQ scores are indicative of higher alexithymic characteristics. PAQ scales demonstrate good internal consistency (Cronbach’s α=0.85 to 0.87) and convergent validity, where PAQ-Total strongly correlates with the Toronto Alexithymia Scale (*r* = 0.76 [74]). PAQ scores are not strongly related to MAIA subscales [100].

### Highly Sensitive Person Scale (HSPS [4])

The HSPS is a 27-item self-report measure of sensory processing sensitivity (SPS), a personality trait involving dispositional heightened processing of external (e.g., light, scent) and internal (e.g., hunger, pain) stimuli. Participants rate items on a 7-point Likert-scale, ranging from *not at all* (1) to *extremely* (7). Whilst SPS was initially conceptualised as unidimensional [4], subsequent research indicates that SPS is composed of three factors: 1) Ease of Excitation (HSPS-EOE), regarding the propensity to feel overwhelmed by external and internal demands, 2) Aesthetic Sensitivity (HSPS-AES), regarding aesthetic awareness, and 3) Low Sensory Threshold (HSPS-LST), involving the tendency to become unpleasantly aroused by external and internal stimuli [87]. As the three-factor solution omits items from the full HSPS, a total score was also calculated, where higher scores indicate heightened SPS. Strong internal consistency has been observed (ɑ=0.89, [87]. The HSPS demonstrates construct validity where EOE and total HSPS scores moderately correlate with Neuroticism (*r* = 0.48 and 0.45, respectively, [87]), SPS appears distinct from interoception, as measured by the BPQ [95].

#### Procedure

Ethics approval was granted by the Victoria University Human Research Ethics Committee (VUHREC). Participants were recruited using the Prolific service, including screening for inclusion/exclusion criteria. Study details were published via Prolific, including the link for the study that was hosted on Qualtrics (Qualtrics, Provo, UT [76]). Participants read through the participant information form detailing the goals and procedures of the study. Then, they reviewed the consent form. Consent was implied by checking the relevant option. Participants provided demographic information and information regarding their general health. Then, they proceeded to the questionnaire battery, consisting of the BMCQ, MAIA, BPQ, BAQ, HSPS, and PAQ. Questionnaires were presented in a randomised order to control for possible ordering effects of responses. In total, the full battery took participants approximately 45 minutes to complete and they were remunerated.

#### Statistical analyses for BMCQ

All analyses were conducted with IBM® SPSS® Statistics (Version 28) [52]. As this study aimed to develop a self-report measure that assesses the degree to which individuals are connected to both body and mind, an exploratory factor analysis (EFA) was employed to substantiate an underlying factor structure, reduce data, and estimate communalities amongst items. To ensure the data were suitable for analysis, all relevant assumptions were assessed. The removal of participants that self-reported a psychiatric diagnosis impacted the envisioned adequate sample size of 400, the included sample of 316 participants with no self-reported psychiatric condition provided a ratio of approximately 14 respondents per item which was deemed sufficient for EFA [11]. Normality amongst items was assessed by observing non-significant skewness values [91]. Univariate linearity was assessed by inspecting scatterplots for each item pairing. As outliers can influence factor solutions, univariate outliers were screened by inspection of histograms for individual items, and multivariate outliers through Mahalanobis’ distance (χ^2^_22_= 48.27, ɑ = .001). To assess the absence of multicollinearity and singularity, squared multiple correlations were assessed. If any of these were 1.00 or close to, the corresponding item would be deleted [91]. Factorability was assessed using several methods. First, intercorrelations amongst items were reviewed, where *r*=>0.32 was required. Bartlett’s Test of Sphericity was also reviewed, with *p* <.05 as the desired parameter. In addition, Kaiser-Meyer-Olin Measure of Sampling Adequacy (KMO) was assessed, with a cut-off of KMO=>0.60 [49]. Diagonals of the anti-image correlation matrix were screened to inform potential item reduction, with a minimum criterion of 0.50 required [91].

To examine the structure of the BMCQ, a combination of techniques was employed, per suggestions from Tabachnick and Fidell [91]. First, a principal components analysis (PCA) with oblique (direct oblimin.) rotation was conducted prior to principal factors extraction analysis to initially estimate the likely number of factors from eigenvalues >1.0 and Scree-plot inspection [19]. Review of the pattern matrix informed item reduction, where loadings <0.32 and/or the presence of complex variables with low primary loadings were indicators for possible item deletion. Next, principal axis factors extraction with oblique rotation (direct oblimin.) was applied to determine the underlying factors as contributing to the body-mind connection construct. This approach was informed by features of principal factors analysis, whereby estimated communalities eliminate unique and error variance from variables, thus providing a salient solution. Oblique rotation (direct oblimin.) was deemed suitable, as items were likely to co-vary [91]. To further determine appropriateness, the factor correlation matrix was examined for correlations around 0.32. Analysis of communalities also informed extraction, where values ≤0.32 were scrutinised and removed. Where variables were complex with cross-loading and low primary loadings (<0.32), these items were also removed [49], 100. Following EFA, retained items were submitted to a reliability analysis. Internal consistency was assessed using Cronbach’s alpha coefficients, where ɑ≥0.70 indicated acceptability.

## Results

### Results of data screening for BMCQ

All relevant assumptions for EFA were assessed. Normality amongst single variables was acceptable. Scatterplot inspection indicated that univariate linearity was acceptable. Whilst no major univariate outliers were detected, 12 multivariate outliers were identified. These cases were omitted from analysis. The remaining sample of 304 participants was deemed sufficient for EFA, as this provided an adequate ratio of approximately 14 respondents per item. No squared multiple correlations were close to 1.00.

### Results of exploratory factor analysis of BMCQ

All 22 BMCQ items were submitted to a preliminary PCA. The initial factorability of the 22 BMCQ items was examined. It was observed that 18 of the 22 items correlated at least *r*=0.32 with at least one other item, indicating adequate factorability. Bartlett’s test of sphericity was significant, *χ*^*2*^_231_= 2430.63, *p* <.001, and KMO measure of sampling adequacy was 0.85. Diagonals of the anti-image correlation matrix were above 0.50. Communalities following extraction indicated that all items were suitable for inclusion, as they exceeded the minimum criterion of 0.32. Scree-plot inspection and Eigenvalues indicated that five to six factors would likely be extracted in the principal factors extraction analysis with all 22 items. Considering these overall indicators, principal factors extraction with oblique rotation was deemed to be suitable with all 22 items.

Principal axis factors extraction with direct oblimin. rotation was then employed. Communality values for five of the 22 items were low, indicating heterogeneity relative to other BMCQ items, alongside indication that these variables were complex with low primary loadings. These items were removed and analyses re-run with 17 items; two items subsequently produced a low communality value and were removed. A four-factor structure was produced with the remaining 15 items. Two items strongly loaded onto a unique factor (Eigenvalue >1.0) with poor internal consistency (ɑ=0.66) and low item-full scale correlations (<0.30). These were subsequently removed and analysis re-run. It was identified that one item cross-loaded (‘*I consider myself in touch with my body and mind’*). Due to this item explicitly concerning mind-body valuation, it was retained for future investigations. Reliability analysis substantiated inclusion, based on consideration of acceptable squared multiple correlation (0.41) and reduced scale reliability, should the item be deleted (0.77 from 0.80; [41]). Accordingly, the BMCQ was reduced from 22 to 13 items. Specifically, the three BMCQ factors explained 39.09%, 8.94%, and 5.99% of variance in the mind-body connection construct, respectively. Table 5 presents factor loadings, communalities, means, and standard deviations for individual items. Variables are ordered, bolded, and grouped by size of loading to facilitate interpretation. Interpretative labels for each factor are included in the table footnote.

**Table 5:** Factor loadings, extracted communalities following principal axis factors extraction with direct oblimin. Rotation for 13 Items from the Body-Mind Connection Questionnaire (BMCQ) with item means and standard deviations (*N=*304)

	Factor					
Item	1^a^	2	3	*M*	*SD*	Extracted Communality
Feeling physically well is something that I prioritise in life.	**0.81**	-0.09	0.02	4.98	1.37	0.60
I value being well-balanced in my body and my mind.	**0.76**	-0.04	-0.04	5.41	1.15	0.50
Feeling mentally well is something that I prioritise in life.	**0.71**	-0.01	0.00	5.33	1.29	0.59
I am usually proactive in addressing the needs of my body.	**0.70**	0.11	0.01	4.65	1.36	0.56
Where possible, I always attend to what my body is telling me.	**0.62**	0.17	-0.03	4.78	1.31	0.52
I feel disconnected from my body. (R)	**0.43**	0.05	-0.29	5.25	1.32	0.43
If I were asked to, I'd find it hard to describe changes in my body associated with positive and negative emotions. (R)	-0.04	**0.83**	0.03	4.41	1.54	0.64
I find it hard to identify changes in my body associated with positive and negative emotions. (R)	-0.09	**0.74**	-0.15	4.65	1.39	0.62
I tend to focus on things happening in my physical environment rather than what is happening inside of me. (R)	0.20	**0.55**	0.05	3.67	1.40	0.40
I can direct my focus toward how specific parts of my body feel.	0.03	0.09	**-0.74**	5.06	1.19	0.64
It is easy for me to focus on specific sensations if they are suddenly experienced.	-0.04	0.04	**-0.71**	5.38	1.02	0.50
It is easy for me to focus on specific sensations if I purposefully think about them.	0.03	-0.07	**-0.70**	5.35	1.08	0.48
I consider myself in touch with my body and mind.	0.36	0.08	**-0.44**	4.99	1.16	0.55

^a^F1: Body-Mind Values, F2: Sensation-Emotion Articulation, F3: Interoceptive Attention

In sum, the three factors of the BMCQ were Body-Mind Values, Sensation-Emotion Articulation, and Interoceptive Attention, reflecting that the body-mind construct involves cognitive and emotional processing of interoceptive signals, and holistic mind-body beliefs. As the scales ranged from three to six items, mean scores for each BMCQ factor were computed for the full sample of 316. As the data were normally distributed, internal consistency reliability and descriptive statistics were evaluated for the full sample with no psychiatric diagnosis (*N=*316). These are presented in Table 6.

**Table 6:** Score interpretation, ranges, descriptive statistics, and cronbach’s alpha coefficients of BMCQ subscales in sample with no psychiatric diagnosis (*N=*316)

Scale	No. of Items	Score Interpretation	Observed Range^a^	*M (SD)*	Skewness	Kurtosis	Alpha	Range of Item-Scale Correlations
Body-Mind Values	6*	Higher values reflect stronger beliefs in mind-body integration and importance of wellbeing.	2.17-7.00	5.06 *(1.01)*	-0.64	0.63	0.85	0.55-0.69
Sensation-Emotion Articulation	3*	Higher values reflect greater internal focus and capacity for articulating bodily changes associated with emotions.	1.00-7.00	4.24 *(1.18)*	-0.26	-0.39	0.74	0.47-0.62
Interoceptive Attention	4	Higher values reflect greater direction of attentional resources toward interoceptive stimuli.	2.00-7.00	5.17 *(0.91)*	-0.42	-0.28	0.8	0.56-0.72

^*^Contains reverse scored items

^a^Possible range from 1-7

Cronbach’s alpha coefficients ranged from 0.74 to 0.85, indicating acceptable to good internal consistency; all item-scale correlations were ≥0.30. Mean scores tended to be high; on a 1-7 scale, means ranged from a low of 4.24 (Sensation-Emotion Articulation) to a high of 5.17 (Interoceptive Attention), indicating that the sample endorsed characteristics underlying a strong connection with mind and body. To further understand the degree to which the BMCQ factors were related, correlations between the scales were conducted and are presented in Table 7.

**Table 7:** Pearson’s correlations between BMCQ scales (*N=*316)

Scale	1	2	3
1. Body-Mind Values	-		
2. Sensation-Emotion Articulation	0.38**	-	
3. Interoceptive Attention	0.59**	0.42**	-

^**^p<.001 (two-tailed)

Plausible directions were observed amongst the subscales, where stronger correlations were observed between theoretically related constructs (e.g., strong positive correlation between Interoceptive Attention and Body-Mind Values), although the magnitude of some relationships indicated distinctness (e.g., Sensation-Emotion Articulation and Body-Mind Values).

Analyses were also undertaken to compare BMCQ scores in demographic variables (age, gender, education level, self-reported BMI, smoking status, sport and exercise engagement, yoga practice, and mindfulness and meditation practice). All means, standard deviations, and statistics are provided in the Supplemental Material. Results indicated that several demographic factors elicited significant differences for several BMCQ scales. These included gender, BMI, sport and exercise engagement, yoga practice, and mindfulness and meditation practice. See Tables S2 and S3 in Supplemental Material.

### Convergent and discriminant validity

For newly developed measures such as the BMCQ, evaluating correlations with other measures sharing theoretical relatedness enables a preliminary understanding of the meaning of the developed scale.

#### Statistical analyses for convergent and discriminant validity

To test for convergent and discriminant validity, a similar approach to Mehling et al. [65] was adopted, by which two integrated analyses of correlational patterns were performed: (1) determining whether the BMCQ scales relate to other measures in ways that are consistent with *a priori* hypotheses, and (2) examining correlations of each BMCQ scale across all validity measures and interpreting the meaning of these relationships (i.e., correlation coefficients >0.30). In presenting the results for convergent and discriminant validity, characteristics across all BMCQ scales in conjunction with validity measures and subscales were interpreted to engender greater clarity regarding the body-mind connection.

#### Hypotheses for correlations between BMCQ and validity measures

*A priori* hypotheses were formulated in terms of strength and directionality. Based on the approach of Mehling et al. [65], the conceptual factor structure of BMCQ and generated items for these domains were reviewed alongside the related constructs. Due to the number of administered validity scales, hypotheses were developed for each individual BMCQ scale using theoretically relevant subsets of validity measures with respect to which scales would be most highly correlated, inclusive of directionality. All correlations were rank ordered according to Cohen’s [22] conventions. This enabled determining whether the measures hypothesised to be most strongly correlated were in the top rank (*r=≥*0.50), mid-rank (*r*=0.30 to 0.49), and low rank (*r*=0.00 to 0.29). Significant correlations were interpreted where *p*<.05 (two-tailed).

The Interoceptive Attention scale assesses spontaneous and purposeful direction of attention toward internal bodily sensations. This was expected to correlate with other measures of mindful, non-biased attentional capacities and tendencies in the context of interoception and bodily awareness (i.e., MAIA, BAQ), but not with scales assessing heightened sensory processing sensitivity (HSPS). Sensation-Emotion Articulation scale items were generated to represent the inverse of alexithymic characteristics, and therefore expected to negatively relate to alexithymia scales, but positively relate to emotional awareness. This was not expected to relate to scores from questionnaires that do not assess the interface of emotion and interoception (i.e., BAQ, BPQ). The Body-Mind Values scale was expected to relate to other scales assessing regulatory and behavioural tendencies involving higher order interoceptive processing and body awareness more strongly (i.e., adaptive MAIA scales, BAQ). It was expected that this would be distinct from scales involving negative emotional interpretations and behaviours related to pain and discomfort. Table 8 presents hypotheses formulated for convergent and discriminant validity.

**Table 8:** Hypothesised correlations between BMCQ Scales and validation measures for convergent and discriminant validity

BMCQ Scale	Convergent Validity	Discriminant Validity
Body-Mind Values	1. Strong positive with MAIA-Trust. 2. Strong positive with MAIA-BL. 3. Moderate positive with MAIA-SR 4. Moderate positive with MAIA-AR 5. Moderate positive with BAQ	1. Weak positive with MAIA-ND 2. Weak positive with MAIA-NW
Sensation-Emotion Articulation	1. Strong negative with PAQ-Total 2. Moderate negative with PAQ-DIF 3. Moderate negative with PAQ-DDF 4. Moderate negative with PAQ-EOT 5. Moderate positive with MAIA-EA	1. Weak positive with BAQ 2. Weak positive with BPQ
Interoceptive Attention	1. Strong positive with MAIA-AR 2. Moderate positive with MAIA-Not. 3. Moderate positive with BAQ	1. Weak positive with HSPS 2. Weak positive with HSPS-EOE 3. Weak positive with HSPS-AES 4. Weak positive with HSPS-LST

Validity Measures: Interoceptive Sensibility and Bodily Awareness: *MAIA-2* Multidimensional Assessment of Interoceptive Awareness, Version 2, *Not* Noticing, *ND* Not-Distracting, *NW* Not Worrying, *AR* Attention Regulation, *EA* Emotional Awareness, *SR* Self-Regulation, *BL* Body Listening, *Trust* Trusting, *BAQ* Body Awareness Questionnaire, *BPQ* Body Perception Questionnaire; Sensory Processing Sensitivity, *HSPS* Highly Sensitive Person Scale, *EOE* Ease of Excitability, *AES* Aesthetic Sensitivity, *LST* Low Sensory Threshold, Alexithymia, *PAQ* Perth Alexithymia Questionnaire, *DIF* Difficulty Identifying Feelings, *DDF* Difficulty Describing Feelings, *EOT* Externally-Oriented Thinking, *PAQ-Total* Global Alexithymia)

#### Results of correlations between BMCQ scales and validity measures

To assess convergent and discriminant validity, correlational analyses were conducted between BMCQ subscales, the MAIA, BPQ, BAQ, HSPS, and PAQ. Results are discussed with respect to each BMCQ scale and hypothesised correlations. Correlations demonstrating a moderate relationship, irrespective of *a priori* hypotheses, are also reported. Table 9 displays correlation coefficients between BMCQ scales and validity measures.

**Table 9:** Pearson’s correlations between BMCQ subscales and validity measures

Validity Measures	Interoceptive Sensibility and Bodily Awareness										Alexithymia				Sensory Processing Sensitivity			
	MAIA-2^a^								BAQ^b^	BPQ^b^	PAQ^a^				HSPS^a^			
BMCQ Subscales	Not.	ND	NW	AR	EA	SR	BL	Trust.			DIF	DDF	EOT	PAQ-Total	EOE	AES	LST	HSPS-Total
Body-Mind Values	0.46**	0.13*	0.06	0.49**	0.42**	0.51**	0.58**	0.67**	0.48**	0.13*	-0.26**	-0.27**	-0.44**	-0.36**	-0.03	0.30**	0.06	0.09
Sensation-Emotion Articulation	0.35**	0.26**	0.02	0.28**	0.33**	0.26**	0.35**	0.27**	0.28**	0.11*	-0.34**	-0.41**	-0.50**	-0.47**	*0.05*	0.22**	0.13*	0.14**
Interoceptive Attention	0.43**	0.14**	0.14*	0.44**	0.34**	0.36**	0.42**	0.52**	0.41**	0.08	-0.37**	-0.37**	-0.46**	-0.45**	-0.01	0.39**	0.04	0.12*

Bolded are the highest correlations for the BMCQ subscale rows, *italicised* are the highest correlations for the validity measure columns

^a^*N=*313

^b^*N=*309 **p<.001, *p<.05 (two-tailed)

Validity Measures: Interoceptive Sensibility and Bodily Awareness: *MAIA-2* Multidimensional Assessment of Interoceptive Awareness, Version 2, *Not* Noticing, *ND* Not-Distracting, *NW* Not Worrying, *AR* Attention Regulation, *EA* Emotional Awareness, *SR* Self-Regulation, *BL* Body Listening, *Trust* Trusting, *BAQ* Body Awareness Questionnaire, *BPQ* Body Perception Questionnaire, Sensory Processing Sensitivity, *HSPS* Highly Sensitive Person Scale, *EOE* Ease of Excitability, *AES* Aesthetic Sensitivity, *LST* Low Sensory Threshold, Alexithymia, *PAQ* Perth Alexithymia Questionnaire, *DIF* Difficulty Identifying Feelings, *DDF* Difficulty Describing Feelings, *EOT* Externally-Oriented Thinking, *PAQ-Total* Global Alexithymia)

##### Body-mind values

Stronger beliefs in mind-body integration and the importance of wellbeing was strongly positively correlated with MAIA-Trust., MAIA-BL, and MAIA-SR scales. Body-Mind Values was moderately positively related to MAIA-AR, and the BAQ. By contrast, Body-Mind Values was marginally positively related to MAIA-ND and did not correlate with MAIA-NW. Additionally, correlational patterns indicated that Body-Mind Values moderately positively correlated with MAIA-Not., MAIA-EA, and HSPS-AES. Additionally, this scale moderately negatively correlated with PAQ-Total and PAQ-EOT.

##### Sensation-emotion articulation

A stronger capacity to identify and describe internal body changes in emotional contexts and be internally focused, as measured by the BMCQ Sensation-Emotion Articulation scale, was moderately negatively correlated with PAQ-Total. Further review of the relationship between Sensation-Emotion Articulation, as measured by the BMCQ, and PAQ alexithymia subscales revealed a strong negative correlation with PAQ-EOT and moderate negative correlations with PAQ-DIF and PAQ-DDF. A moderate positive correlation with MAIA-EA was also observed. There were small but significant positive correlations between Sensation-Emotion Articulation and the BAQ and BPQ, respectively. Correlational patterns indicated that Sensation-Emotion Articulation was also moderately positively correlated with MAIA-Not. and MAIA-BL.

##### Interoceptive attention

The BMCQ Interoceptive Attention scale, which captures the self-reported ability to direct attentional resources toward internal bodily sensations showed moderate positive correlations with MAIA-AR, MAIA-Not., and the BAQ. A low positive correlation with HSPS-Total was observed; Interoceptive Attention was not significantly correlated with HSPS-EOE or HSPS-LST. However, there was a moderate positive correlation with HSPS-AES. In examining patterns of correlations (*r* ≥0.30), Interoceptive Attention strongly positively correlated with MAIA-Trust., moderately positively correlated with MAIA-BL and MAIA-SR, and moderately negatively correlated with PAQ-Total, PAQ-DIF, PAQ-DDF, and PAQ-EOT. See Table 9.

## Discussion

Increasing interest in perceptions and characteristics underlying the mind-body connection has prompted the need for reliable, valid, and efficient measures. The BPQ, MAIA, and BAQ are well established and widely used in the assessment of IS [35]. Although these measures were not developed for the specific purpose of assessing the mind-body connection, interoceptive self-report scales have served as primary assessment tools in mind-body research [75] and as outcome measures to evaluate the effects psychological interventions aimed at improving mind-body integration (e.g., [14]). The aim of this research was to develop a new self-report measure that explicitly assesses the mind-body connection: the BMCQ. This study describes the development and preliminary psychometric evaluation of the BMCQ. An EFA resulted in a three-factor solution reflecting relatively distinct factors underpinning one’s connection with both body and mind: Body-Mind Values, Sensation-Emotion Articulation, and Interoceptive Attention.

### Internal consistency

The Body-Mind Values, Sensation-Emotion Articulation, and Interoceptive Attention scales produced acceptable to good internal consistency, as demonstrated by coefficient alpha, which were 0.85, 0.74, and 0.80, respectively. Coefficients are contingent upon the number of constituent items, which can explain why the Sensation-Emotion Articulation scale—comprised of three items—produced the lowest. Despite generating positively- and negatively worded items for this scale, the EFA led to the deletion of positively worded items generated for this construct. As such, the scale consisted of only negatively worded items which could have contributed to the coefficient obtained in this present preliminary psychometric assessment [41]. Despite this, BMCQ scales should be considered relative to other established multidimensional questionnaires assessing similar constructs. These present findings reflect ranges observed for theoretically associated MAIA scales in initial [65] and subsequent validations. This indicates that the BMCQ is a reliable measure of mind-body connection domains, despite the scales containing substantially fewer items.

### Convergent and discriminant validity

As this is a newly developed self-report measure of the mind-body connection, preliminarily examining convergent and discriminant validity was of utmost importance. Although metrics for establishing convergent and discriminant validity vary across the literature and in practice [20], the method employed in the present study enabled identification of convergent and discriminant evidence for the BMCQ. Expected correlations were hypothesised and generally supported by the data from the present sample. Collectively, the results highlight that BMCQ scales reflect distinct perceptions, as they conceptually and empirically differ to pre-existing tools that measure theoretically associated constructs, and that the mind-body connection should be regarded as a multidimensional construct.

#### Body-mind values

The Body-Mind Values scale was designed to be a more explicit measure of embodied views and holistic wellbeing. Mind-body beliefs, as measured by this BMCQ scale, were strongly positively correlated with the experience of one’s body as safe and trustworthy (MAIA-Trust.), active listening to the body for insight (MAIA-BL) and regulating distress by attention to body sensations (MAIA-SR). These three scales arguably represent the zenith of healthy mind-body integration [65]. Experiencing the body as safe with sufficient trust instilled in one’s interpretation of sensations guides motivated decision-making in both the present and future, as informed by one’s high-level model that comprises past experiences (e.g., [5, 83]). Substantiating this, moderate positive correlations were observed between the Body-Mind Values scale and MAIA-AR and the BAQ. In terms of implications for motivation and behaviour, the capacity to purposefully focus on the body, regulate attention and distress, and gain additional insight about emotional states to enhance the precision of sensation interpretation [14] may serve as the behavioural outcomes of stronger views regarding mind-body integration and wellbeing importance. Considered together, these related measures capture interoceptive and emotional processing, regulation, and goal-directed action. Furthermore, reviewed correlational patterns revealed moderate positive relationships between Beliefs and Behaviours and measures encapsulating adaptive attentional and regulatory styles involving interoception [63], specifically in awareness of uncomfortable, comfortable, and neutral body sensations (MAIA-Not.) and the connection between body sensations and emotional states (MAIA-EA), which reflect functional interoception [35]. Furthermore, Body-Mind Values was moderately negatively correlated with global alexithymia (PAQ-Total), but particularly in terms of externally oriented thinking and preferences for focussing on the external environment (PAQ-EOT). Taken together, the Body-Mind Values scale encompasses holistic beliefs underscored by a healthy capacity to connect body with mind and facilitates adaptivity in the face of environmental challenges. Thus, it is tenable that these interoceptive and affective concepts underpin one’s broader Body-Mind Values, as conceptualised by the BMCQ.

Discriminant evidence further suggested that the Body-Mind Values scale is distinct from the tendency to not ignore or distract oneself from sensations of pain or discomfort (MAIA-ND) or experience minimal emotional distress or worry (MAIA-NW). This is in line with previous findings which demonstrated these MAIA scales are related to pain catastrophising and maladaptive bodily awareness. However, it is noted that the present study excluded participants with chronic pain conditions that might underlie altered attentional focus and catastrophising [13].

#### Sensation-emotion articulation

The Sensation-Emotion Articulation scale was created to assess the capacity to articulate the emotional meaning of internal bodily sensations, in conjunction with internally oriented thinking. As anticipated, moderate to strong correlations were observed with difficulties identifying feelings (PAQ-DIF), difficulties describing feelings (PAQ-DDF), externally oriented thinking (PAQ-EOT), and global alexithymia (PAQ-Total), as was a moderate positive correlation with MAIA-EA. Such capacities could underpin capabilities for cultivating emotions with precision and specificity across multiple contexts [7, 96]. These abilities are purported to be founded on an unambiguous identification and articulation of feelings [51], which characterises the ability to connect mind with body. Furthermore, the moderate correlation with MAIA-BL might further suggest that the ability to articulate emotions, based on interpretation of bodily sensations, is important for situationally appropriate, motivated behaviour. Together, this implies that the BMCQ capably measures more complex emotional processing of bodily factors than existing self-reports that are used in mind-body research. This is particularly evident when considering other lines of evidence which are weakly suggestive of an association between aspects of self-reported interoception and the multiple facets of alexithymia (e.g., [46, 96]). Though the MAIA-EA scale arguably assesses the ability to identify feelings through connection of sensations with emotions, the present findings demonstrate stronger relationships with alexithymic traits not captured by other scales conceptualised as measures of the mind-body connection—traits that typify a maladaptive mind-body disconnection [31]. Promisingly, the BMCQ Sensation-Emotion Articulation scale explicitly captures these capacities.

This view is particularly emphasised upon review of discriminant evidence for the Sensation-Emotion Articulation scale. Although they were significant, correlations with the BAQ and BPQ were observed to be small in magnitude. Accordingly, this scale is distinct from attentiveness to normal non-emotive body processes (BAQ) and maladaptive hypersensitivity toward bodily sensations (BPQ), given that each of these measures do not examine the interface of interoception and emotion [64]. Moreover, further review of correlations between Sensation-Emotion Articulation and other MAIA subscale scores generally substantiates the view that the scale captures functions beyond facets of subjective interoceptive awareness.

#### Interoceptive attention

The Interoceptive Attention scale was designed to operationalise the notion that attention can be purposefully and spontaneously directed toward internal bodily sensations. As expected, Interoceptive Attention scores showed moderate positive correlations with MAIA-AR, MAIA-Not., and the BAQ. Each of these scales assess elements of self-reported attentional processes, ranging in complexity. MAIA-Not. forms a foundational facet of attention, by which baseline awareness of sensations is measured. By contrast, MAIA-AR qualitatively involves more sophisticated body-centric attentional processes (i.e., sustained, divided attention), whereas BAQ items involve a capacity to integrate external information relative to the physiological condition of the body [35]. In line with the present correlational evidence, the Interoceptive Attention scale can be interpreted as a parsimonious self-report measure of interoceptive attentional control. Un-hypothesised patterns of correlations further revealed moderate to strong correlations between Interoceptive Attention and measures encapsulating adaptive attentional and regulatory styles involving interoception [63], including more active listening to the body for insight (MAIA-BL) and greater experiences of one's body as safe and trustworthy (MAIA-Trust.), as well as lower global alexithymia (PAQ-Total)—particularly a diminished preference for features of the external environment (PAQ-EOT). Collectively, this suggests that stronger interoceptive attentional control, as measured by Interoceptive Attention, may facilitate sensing and addressing ‘somatic markers’ [28] when affective appraisals of bodily sensations are trusted. This is particularly salient, given increasing acknowledgement that bodily states arising from homeostatic perturbation serve as a critical factor in increasing attention, which, in turn, motivates situationally appropriate behaviour to address perceived bodily needs and thus expedite equilibrium [24, 30, 33].

Discriminant validity was also demonstrated, as the Interoceptive Attention scale was mostly unrelated to sensory processing sensitivity—a trait typified by deep cognitive processing of external and internal stimuli that is augmented by greater negative emotional reactivity [3]. Specifically, there was a small positive correlation between Interoceptive Attention and global sensory processing sensitivity (HSPS-Total), and no significant correlation with the propensity to feel overwhelmed by external and internal demands (HSPS-EOE) or experiencing unpleasant arousal in processing of internal and external stimuli (HSPS-LST). Given that such sensory processing characteristics appear driven by avoidance of negative consequences and unpleasant states, and high distractibility [87], this lack of relationship is feasible as the present sample demonstrated non-judgemental, adaptive processing of internal bodily sensations [63]. This is corroborated by the observed small correlation between Interoceptive Attention and the BPQ—a measure of maladaptive attention toward internal bodily sensations [63], and MAIA-ND and MAIA-NW scales, which relate more strongly to pain catastrophising and anxiety-driven bodily focus. In sum, the present results lend credence to the view that Interoceptive Attention forms the basis of the mind-body connection.

## Limitations

While the findings are valuable, several limitations should be considered. The convenience sampling, cross-sectional design, and reliance on self-report data for the measurement of these constructs indicate that there should be some caution exerted in interpreting results. This is pertinent, given the proportion of the sample that provided demographical information that has been related to poorer health and wellbeing outcomes (e.g., self-reported BMI, psychiatric diagnosis). Furthermore, it is acknowledged that one item was retained as part of the Interoceptive Attention scale, despite cross-loading on the scale it was generated for—Body-Mind Values. Accordingly, cross-validation studies are strongly suggested to further confirm the structure and validity of the BMCQ. Furthermore, the present study recruited 101 individuals that self-reported a range of psychiatric diagnosis that can differentially affect mind-body connection perceptions (e.g., anxiety and depression; [56]). Samples within the disorder categories were deemed insufficiently powered to interpret EFA of BMCQ factor structures for different disorders. It is suggested that future studies consider more purposeful sampling of clinical populations, coupled with measurement of disorder symptomatology that enables identification of severity, such as administering validated screening tools. This will enable greater clarification of whether the mind-body connection is conceptualised differently amongst particular clinical samples, based on whether the three-factor structure is replicated and further, whether correlational patterns differ.

### Implications and future directions

Despite these factors, the current findings are of clinical relevance, considering arguments for the importance of coaching individuals with alexithymia to utilise awareness-of-sensation practices, so as to enable more granular differentiation between bodily perceptions and psychological interpretation [84], the effects of which could be measured utilising the BMCQ scales. A multitude of psychiatric disorders are now viewed as involving both interoceptive and emotional dysfunctions [8, 12, 55, 69]. There appears to be an emergent consideration of interoception in the development of therapeutic interventions that seek to target facets of IS in order to enhance global bodily awareness (e.g., MAIA domains, [14]. However, existing interoceptive self-report measures and interventions drawing upon such features may engender identification of sensations or establishing a baseline connection between sensations and emotions. Although functions such as listening to one’s body for insight and bodily trust are important bases for connecting body with mind, the present findings suggest that articulating the link between sensations and emotions, as well as values regarding mind-body integration and wellbeing, are relevant factors. Such appraisals and values may further influence how individuals adapt to and cope with environmental stressors and challenges.

The BMCQ therefore presents a promising tool for clinicians that could also enable the development of more targeted psychological interventions. For instance, clinical populations such as those typified by comorbid interoceptive and emotional dysfunction (e.g., somatic symptom disorder; [50, 56]) could greatly benefit from treatments drawing upon BMCQ constituents. Interventions could potentially address characteristic externalisation that contributes to maladaptive misattributions of bodily sensations across contexts. In doing so, future work should seek to elucidate how Interoceptive Attention, Sensation-Emotion Articulation, and Body-Mind Values factors interact and link with wellbeing outcomes. Whilst the conceptualisation phase of scale development identified three factors that could be targeted in mind-body therapies, it would be valuable to understand whether these mechanisms are hierarchical and relate to an overall second-order construct. Such findings could support the delivery of interventions that are tailored to the presenting individual.

In conclusion, the results from this study indicate that the BMCQ is a parsimonious measure of the mind-body connection. The self-report may be a valuable tool for assessing perceptions related to one’s concept of mind-body constituents. To our knowledge, the BMCQ is the first self-report measure that incorporates interoceptive attentional control, the ability to articulate the link between sensations and emotions, and broader mind-body values. Furthermore, the BMCQ is a brief, convenient 13-item self-report measure that extends upon elements of the mind-body connection disparately captured by other self-report scales. The BMCQ provides researchers and clinicians with an alternative Interoceptive Attention scale that differs from pre-existing tools that capture the proclivity to notice sensations, because it considers the direction and allocation of resources to bodily sensations in spontaneous and purposeful manners. Furthermore, the Sensation-Emotion Articulation scale builds upon how emotion is currently captured in similar scales, where awareness of the connection between sensations and discrete emotions is emphasised. With this new scale, identifying and describing this connection is assessed, alongside preference for an internally oriented focus, which is inversely related to alexithymia. Lastly, the BMCQ provides an alternative scale of mind-body beliefs than is currently captured in existing tools; broader, albeit explicit, measurement of whether mind and body are viewed as integrated entities is now captured by the BMCQ. Though the Body-Mind Values scale strongly related to body listening and trusting, endorsement of such concepts may underlie the value one places on their holistic wellbeing.

The present findings indicate that the BMCQ serves as a basis for considering Sensation-Emotion Articulation and Body-Mind Beliefs as forming part of one’s mind-body connection in addition to Interoceptive Attention. Based on the preliminary assessment of construct validity, the BMCQ should be considered a multidimensional self-report measure of the mind-body connection—not subjective interoception. Accordingly, the scales and constructs comprising the BMCQ could better serve research pursuits pertaining to the mind-body connection and importantly enable the development of targeted psychological interventions aimed at fostering and fortifying adaptive mind-body connections.

### Supplementary Information

**Additional file 1: Table S1. **Self-Disclosed Diagnoses of Physical and Psychiatric Conditions. **Table S2. **Means and Standard Deviations for BMCQ Scales According to Age, Education Level, BMI (Self-Reported), Smoking Status, Alcohol Consumption, Psychiatric Diagnosis, Sport and Exercise Engagement, Yoga Practice, and Mindfulness and Meditation Practice (*N*=316). **Table S3. **Results of Group Difference Analyses for BMCQ Scales According to Age, Education Level, BMI (Self-Reported), Smoking Status, Alcohol Consumption, Psychiatric Diagnosis, Sport and Exercise Engagement, Yoga Practice, and Mindfulness and Meditation Practice (*N*=316).

## Data Availability

Data will be made available upon request.
