# Supplementary material for: Assessment of the mind-body connection: preliminary psychometric evidence for a new self-report questionnaire
Source: BMC Psychol. 2023 Oct 6;11:309. doi: 10.1186/s40359-023-01302-3 (PMC10557351; doi:10.1186/s40359-023-01302-3)
Supplement: Supplementary file 1 — Additional file 1: Table S1. Self-Disclosed Diagnoses of Physical and Psychiatric Conditions. Table S2. Means and Standard Deviations for BMCQ Scales According to Age, Education Level, BMI (Self-Reported), Smoking Status, Alcohol Consumption, Psychiatric Diagnosis, Sport and Exercise Engagement, Yoga Practice, and Mindfulness and Meditation Practice (N=316). Table S3. Results of Group Difference Analyses for BMCQ Scales According to Age, Education Level, BMI (Self-Reported), Smoking Status, Alcohol Consumption, Psychiatric Diagnosis, Sport and Exercise Engagement, Yoga Practice, and Mindfulness and Meditation Practice (N=316). [file 40359_2023_1302_MOESM1_ESM.docx]

**Physical and Psychiatric Diagnoses Self-Reported by Participants**

Participants who nominated that they had a current diagnosis of a physical and/or mental condition were prompted to disclose of their diagnoses using a free-text option. These were manually reviewed by the first author. For physical diagnoses, these were categorised according to the bodily system affected where applicable. For psychiatric diagnoses, clinical categorisations were made according to disorder categories included in the Diagnostic and Statistical Manual of Mental Disorders (5th ed.; DSM–5; American Psychiatric Association, 2013), and were not derived from formal clinical diagnoses or interviews. Table S1 contains current conditions that were self-reported by 119 participants.

| **Table S1.** *Self-Disclosed Diagnoses of Physical and Psychiatric Conditions* | |
| --- | --- |
| Diagnosis | *N* (% of cases) **^a^** |
| **Physical Diagnosis** | 18 (4.32%) |
| Autoimmune (e.g., hypo-, hyperthyroidism) | 3 (16.7%) |
| Cardiovascular | 1 (5.6%) |
| Gastrointestinal | 1 (5.6%) |
| Gynaecological | 2 (11.1%) |
| Long COVID | 1 (5.6%) |
| Neurological (e.g., migraine) | 2 (11.1%) |
| Ocular | 1 (5.6%) |
| Respiratory (e.g., asthma) | 7 (38.9%) |
| **Psychiatric Diagnosis** | 101 (24.2%) |
| Anxiety Disorders | 60 (59.4%) |
| Bipolar and Related Disorders | 4 (4.0%) |
| Depressive Disorders | 53 (52.5%) |
| Feeding and Eating Disorders | 2 (2.0%) |
| Neurodevelopmental Disorders | 19 (18.8%) |
| Obsessive-Compulsive and Related Disorders | 10 (9.9%) |
| Personality Disorders | 2 (2.0%) |
| Sleep-Wake Disorders | 1 (1.0%) |
| Somatic Symptom and Related Disorders | 1 (1.0%) |
| Trauma- and Stressor-Related Disorders | 7 (6.9%) |
| Not Specified | 1 (1.0%) |
| **Comorbid Psychiatric Disorders^b^** | **47 (46.5%)** |
| **Comorbid Physical and Psychiatric Diagnoses** | **8 (1.9%)** |
| **^a^** Percentage exceeds 100%, for psychiatric diagnosis, as multiple participants reported two or more diagnoses.  ^b^ Anxiety comorbid to Depression (*n*=21), Anxiety comorbid to Depression and other disorders (e.g., Obsessive-Compulsive; *n*=10), Anxiety comorbid to another disorder (e.g., OCD, *n*=7), Depression comorbid to another disorder (e.g., ADHD, *n*=2), Bipolar and Related comorbid to another disorder (e.g., Feeding and Eating; *n*=2), Neurodevelopmental disorder comorbid to another disorder (e.g., Personality Disorder; *n*=4). | |

**Body-Mind Connection Questionnaire Administered for Field Testing**

# Body-Mind Connection Questionnaire

This section asks you to indicate how applicable a series of statements regarding your body and mind are to you generally. By that, we mean how they apply to you most of the time. Some of these will be a series of statements related to bodily sensations (e.g., hunger, thirst, need for air, etc.) and/or emotions. There are no right or wrong answers.

| 1. I consider myself in touch with my body and mind. | 1  Very untrue of me | 2  Untrue of me | 3  Somewhat untrue of me | 4  Neutral | 5  Somewhat true of me | 6  True of me | 7  Very true of me |
| --- | --- | --- | --- | --- | --- | --- | --- |
| 1. I can direct my focus toward how speciﬁc parts of my body feel. | 1  Very untrue of me | 2  Untrue of me | 3  Somewhat untrue of me | 4  Neutral | 5  Somewhat true of me | 6  True of me | 7  Very true of me |
| 1. It is easy for me to focus on speciﬁc sensations if they are suddenly experienced. | 1  Very untrue of me | 2  Untrue of me | 3  Somewhat untrue of me | 4  Neutral | 5  Somewhat true of me | 6  True of me | 7  Very true of me |
| 1. It is easy for me to focus on speciﬁc sensations if I purposefully think about them. | 1  Very untrue of me | 2  Untrue of me | 3  Somewhat untrue of me | 4  Neutral | 5  Somewhat true of me | 6  True of me | 7  Very true of me |
| 1. I often push my bodily sensations to run in the background when I am busy. * | 1  Very untrue of me | 2  Untrue of me | 3  Somewhat untrue of me | 4  Neutral | 5  Somewhat true of me | 6  True of me | 7  Very true of me |
| 1. If I have not thought about my bodily sensations for some time, it is challenging for me to become aware of them again. * | 1  Very untrue of me | 2  Untrue of me | 3  Somewhat untrue of me | 4  Neutral | 5  Somewhat true of me | 6  True of me | 7  Very true of me |
| 1. Where possible, I always attend to what my body is telling me. | 1  Very untrue of me | 2  Untrue of me | 3  Somewhat untrue of me | 4  Neutral | 5  Somewhat true of me | 6  True of me | 7  Very true of me |
| 1. I am usually proactive in addressing the needs of my body. | 1  Very untrue of me | 2  Untrue of me | 3  Somewhat untrue of me | 4  Neutral | 5  Somewhat true of me | 6  True of me | 7  Very true of me |
| 1. I don’t generally experience emotions alongside bodily changes (e.g., changes in heartrate or breathing, sweating). * | 1  Very untrue of me | 2  Untrue of me | 3  Somewhat untrue of me | 4  Neutral | 5  Somewhat true of me | 6  True of me | 7  Very true of me |
| 1. I generally experience bodily changes alongside emotions. | 1  Very untrue of me | 2  Untrue of me | 3  Somewhat untrue of me | 4  Neutral | 5  Somewhat true of me | 6  True of me | 7  Very true of me |
| 1. I’m not really concerned about how bodily sensations make me feel. * | 1  Very untrue of me | 2  Untrue of me | 3  Somewhat untrue of me | 4  Neutral | 5  Somewhat true of me | 6  True of me | 7  Very true of me |
| 1. I find it hard to *identify* changes in my body associated with positive or negative emotions. * | 1  Very untrue of me | 2  Untrue of me | 3  Somewhat untrue of me | 4  Neutral | 5  Somewhat true of me | 6  True of me | 7  Very true of me |
| 1. If I were asked to, I’d find it hard to *describe* changes in my body associated with positive or negative emotions. * | 1  Very untrue of me | 2  Untrue of me | 3  Somewhat untrue of me | 4  Neutral | 5  Somewhat true of me | 6  True of me | 7  Very true of me |
| 1. I tend to focus on things happening in my physical environment rather than what is happening inside of me. * | 1  Very untrue of me | 2  Untrue of me | 3  Somewhat untrue of me | 4  Neutral | 5  Somewhat true of me | 6  True of me | 7  Very true of me |
| 1. I often forget to drink unless there is a drink readily at hand. * | 1  Very untrue of me | 2  Untrue of me | 3  Somewhat untrue of me | 4  Neutral | 5  Somewhat true of me | 6  True of me | 7  Very true of me |
| 1. I eat at mealtimes, regardless of whether I’m hungry. * | 1  Very untrue of me | 2  Untrue of me | 3  Somewhat untrue of me | 4  Neutral | 5  Somewhat true of me | 6  True of me | 7  Very true of me |
| 1. I listen to my body to decide when to stop eating or drinking after being very hungry or thirsty. | 1  Very untrue of me | 2  Untrue of me | 3  Somewhat untrue of me | 4  Neutral | 5  Somewhat true of me | 6  True of me | 7  Very true of me |
| 1. After eating a main meal, I typically experience both a sense of fullness and a change in my emotions. | 1  Very untrue of me | 2  Untrue of me | 3  Somewhat untrue of me | 4  Neutral | 5  Somewhat true of me | 6  True of me | 7  Very true of me |
| 1. I feel disconnected from my body. * | 1  Very untrue of me | 2  Untrue of me | 3  Somewhat untrue of me | 4  Neutral | 5  Somewhat true of me | 6  True of me | 7  Very true of me |
| 1. Feeling physically well is something that I prioritise in life. | 1  Very untrue of me | 2  Untrue of me | 3  Somewhat untrue of me | 4  Neutral | 5  Somewhat true of me | 6  True of me | 7  Very true of me |
| 1. Feeling mentally well is something that I prioritise in life. | 1  Very untrue of me | 2  Untrue of me | 3  Somewhat untrue of me | 4  Neutral | 5  Somewhat true of me | 6  True of me | 7  Very true of me |
| 1. I value being well-balanced in my body and my mind. | 1  Very untrue of me | 2  Untrue of me | 3  Somewhat untrue of me | 4  Neutral | 5  Somewhat true of me | 6  True of me | 7  Very true of me |

Asterisks indicate reverse scoring of item.

**Table S2**

*Means and Standard Deviations for BMCQ Scales According to Age, Education Level, BMI (Self-Reported), Smoking Status, Alcohol Consumption, Psychiatric Diagnosis, Sport and Exercise Engagement, Yoga Practice, and Mindfulness and Meditation Practice (N=316).*

|  |  | Interoceptive Attention | |  | Sensation-Emotion Articulation | |  | Body-Mind Beliefs | |
| --- | --- | --- | --- | --- | --- | --- | --- | --- | --- |
| Characteristic | *n* | *M* | *SD* |  | *M* | *SD* |  | *M* | *SD* |
| Age |  |  |  |  |  |  |  |  |  |
| 18-19 | 28 | 5.04 | 0.66 |  | 4.11 | 1.12 |  | 4.82 | 1.09 |
| 20-29 | 119 | 5.15 | 0.99 |  | 4.20 | 1.22 |  | 5.08 | 1.03 |
| 30-39 | 117 | 5.17 | 0.95 |  | 4.28 | 1.22 |  | 5.17 | 1.02 |
| 40-50 | 51 | 5.25 | 0.69 |  | 4.27 | 1.06 |  | 4.90 | 0.87 |
| Gender |  |  |  |  |  |  |  |  |  |
| Male | 124 | 5.15 | 0.87 |  | 3.98 | 1.08 |  | 5.15 | 0.96 |
| Female | 189 | 5.17 | 0.93 |  | 4.40 | 1.22 |  | 5.01 | 1.03 |
| Education |  |  |  |  |  |  |  |  |  |
| Year 10 or lower | 6 | 5.04 | 1.02 |  | 3.61 | 1.25 |  | 4.81 | 0.63 |
| Year 12 | 121 | 5.04 | 0.94 |  | 4.10 | 1.19 |  | 4.99 | 1.12 |
| Bachelor’s degree | 111 | 5.22 | 0.92 |  | 4.26 | 1.23 |  | 5.07 | 0.99 |
| Honours | 15 | 5.20 | 0.71 |  | 4.31 | 1.17 |  | 5.40 | 0.61 |
| TAFE or vocational training | 11 | 5.30 | 0.51 |  | 4.27 | 1.08 |  | 5.11 | 0.68 |
| Masters | 41 | 5.21 | 0.95 |  | 4.57 | 1.07 |  | 5.09 | 1.00 |
| PhD or Doctorate | 6 | 5.58 | 0.74 |  | 4.17 | 0.81 |  | 5.36 | 0.55 |
| Graduate Certificate | 5 | 5.90 | 0.45 |  | 4.93 | 1.19 |  | 5.33 | 1.06 |
| BMI (Self-Report) |  |  |  |  |  |  |  |  |  |
| Underweight | 10 | 5.43 | 0.53 |  | 4.83 | 0.89 |  | 4.97 | 0.62 |
| Normal | 136 | 5.31 | 0.86 |  | 4.36 | 1.14 |  | 5.27 | 1.04 |
| Overweight | 76 | 4.95 | 1.09 |  | 3.98 | 1.28 |  | 4.94 | 1.05 |
| Obese | 74 | 5.07 | 0.83 |  | 4.23 | 1.09 |  | 4.80 | 0.91 |
| Smoking Status |  |  |  |  |  |  |  |  |  |
| Smoker | 33 | 5.07 | 1.19 |  | 3.94 | 1.41 |  | 4.78 | 1.27 |
| Non-smoker | 283 | 5.18 | 0.87 |  | 4.27 | 1.15 |  | 5.10 | 0.97 |
| Alcohol Consumption |  |  |  |  |  |  |  |  |  |
| 0-1 times per week | 224 | 5.19 | 0.90 |  | 4.27 | 1.19 |  | 5.08 | 1.01 |
| 1-2 times per week | 56 | 5.16 | 0.88 |  | 4.10 | 1.18 |  | 5.14 | 1.00 |
| 2-3 times per week | 19 | 5.17 | 0.86 |  | 4.56 | 1.08 |  | 5.26 | 0.78 |
| 3-4 times per week | 7 | 5.29 | 0.94 |  | 3.81 | 1.29 |  | 4.52 | 1.05 |
| 4 or more times per week | 10 | 4.53 | 1.26 |  | 3.93 | 1.09 |  | 4.25 | 1.01 |
| Sport and Exercise |  |  |  |  |  |  |  |  |  |
| Yes | 208 | 5.27 | 0.85 |  | 4.26 | 1.18 |  | 5.26 | 0.93 |
| No | 108 | 4.96 | 0.98 |  | 4.19 | 1.19 |  | 4.69 | 1.06 |
| Yoga |  |  |  |  |  |  |  |  |  |
| Yes | 52 | 5.50 | 0.80 |  | 4.67 | 1.05 |  | 5.57 | 0.82 |
| No | 264 | 5.10 | 0.91 |  | 4.15 | 1.19 |  | 4.96 | 1.01 |
| Mindfulness and Meditation |  |  |  |  |  |  |  |  |  |
| Yes | 75 | 5.55 | 0.81 |  | 4.49 | 1.24 |  | 5.54 | 0.80 |
| No | 241 | 5.04 | 0.90 |  | 4.16 | 1.15 |  | 4.91 | 1.02 |

**Table S3**

*Results of Group Difference Analyses for BMCQ Scales According to Age, Education Level, BMI (Self-Reported), Smoking Status, Alcohol Consumption, Psychiatric Diagnosis, Sport and Exercise Engagement, Yoga Practice, and Mindfulness and Meditation Practice (N=316).*

|  | Interoceptive Attention | | | |  | Sensation-Emotion Articulation | | | |  | Body-Mind Values | | | |
| --- | --- | --- | --- | --- | --- | --- | --- | --- | --- | --- | --- | --- | --- | --- |
| Characteristic | *df* | Statistic | *p* | Group Comparisons |  | *df* | Statistic | *p* | Group Comparisons |  | *df* | Statistic | *p* | Group Comparisons |
| Age | 3, 108.47 | 0.60 | .616 |  |  | 3, 99.82 | 0.24 | .868 |  |  | 3, 311 | 1.47 | .228 |  |
| Gender | 311 | -0.22 | .829 |  |  | 311 | -3.07 | .002 | Females > Males |  | 311 | 1.18 | .239 |  |
| Education | 7, 308 | 1.11 | .356 |  |  | 7, 308 | 1.68 | .298 |  |  | 7, 308 | 0.51 | .828 |  |
| BMI (Self-Report) | 3, 292 | 3.06 | .029 | Normal > Overweight |  | 3, 292 | 2.67 | .048 |  |  | 3, 292 | 4.04 | .008 | Normal > Obese |
| Smoking Status | 36.09 | -0.51 | .615 |  |  | 314 | -1.53 | .126 |  |  | 36.47 | -1.37 | .180 |  |
| Alcohol Consumption | 4, 311 | 1.33 | .260 |  |  | 4, 311 | 0.99 | .418 |  |  | 4, 311 | 2.46 | .046 |  |
| Sport and Exercise | 314 | 2.90 | .004 | Regular Sport > No Regular Sport |  | 314 | 0.56 | .573 |  |  | 314 | 4.9 | <.001 | Regular Sport > No Regular Sport |
| Yoga | 314 | 2.99 | .003 | Yoga > No Yoga |  | 314 | 2.89 | .004 | Yoga > No Yoga |  | 84.55 | 4.06 | <.001 | Yoga > No Yoga |
| Mindfulness and Meditation | 314 | 4.36 | <.001 | Mindfulness > No Mindfulness |  | 314 | 2.13 | .035 | Mindfulness > No Mindfulness |  | 156.93 | 5.55 | <.001 | Mindfulness > No Mindfulness |

Adjusted degrees of freedom are reported where homogeneity of variance was not assumed (Levene’s *p*<.05). For post-hoc comparisons, Bonferroni correction was interpreted; Games-Howell interpreted for non-parametric F-test (Welch’s).
